# Patient delay in cancer studies: a discussion of methods and measures

**DOI:** 10.1186/1472-6963-9-189

**Published:** 2009-10-19

**Authors:** Rikke Sand Andersen, Peter Vedsted, Frede Olesen, Flemming Bro, Jens Søndergaard

**Affiliations:** 1 The Research Unit for General Practice, Institute of Public Health, University of Aarhus, Bartholins Allé 2, DK-8000 Aarhus C, Denmark; 2 Department of Family Medicine, Institute of Public Health, University of Aarhus, Bartholins Allé 2, DK-8000 Aarhus C, Denmark; 3 The Research Unit for Family Medicine, Institute of Health Services Research, University of Southern Denmark, JB Windsløws Vej 9A, DK-5000 Odense C, Denmark

## Abstract

**Background:**

There is no validated way of measuring the prevalence and duration of patient delay, and we do not know how people perceive and define the time intervals they are asked to report in patient delay studies. This lack of a validated measure hampers research in patient delay and is counterproductive to efforts directed at securing early diagnosis of cancer.

**Discussion:**

The main argument of the present paper is that current studies on patient delay do not sufficiently consider existing theories on symptom interpretation. It is illustrated that the interpretation of bodily sensations as symptoms related to a specific cancer diagnosis is embedded within a social and cultural context. We therefore cannot assume that respondents define delay periods in identical ways.

**Summary:**

In order to improve the validity of patient delay studies, it is suggested that research be strengthened on three counts: More research should be devoted to symptom interpretation processes, more research should seek to operationalise patient delay, and, importantly, more research is needed to develop valid instruments for measuring patient delay.

## Background

Delay in general and patient delay in particular has been an issue of concern within health services research for decades [1-4]. Pack and Gallo introduced the concept in 1938, and they defined it as 'an interval between the onset of symptoms and the first visit to a physician'. Undue delay was arbitrarily defined as three months or more [2]. Although patient delay has been recognized for years, many methodologically relevant dimensions of this concept remain unresolved. Only a few studies have examined the reliability and validity of patient delay measures, [5,6] and we lack thorough knowledge on how people perceive and define the time intervals they are asked to report.

In cancer diagnosis, studies indicate that it is possible to improve the prognosis and reduce the need for extensive and aggressive treatment if cancer is detected at an early stage [7,8]. However, the lack of a validated measure of patient delay makes it difficult to properly investigate the causes of patient delay and to establish the contribution of patient delay to the total diagnostic delay, and its effect on morbidity and mortality. Thus, we need to better understand what we are actually measuring in terms of patient delay, a need which is underscored by the contradictory and diverging results of existing patient delay studies [9,10].

The aim of this paper is to initiate a debate on the methodological problems related to measuring patient delay in cancer studies. It is argued that current studies of patient delay do not sufficiently incorporate existing theories on symptom interpretation, but often adopt a simplistic, empiricist view on how people interpret symptoms. In order to improve measures of patient delay, we need to integrate theories that acknowledge more complex aspects of how people interpret and recognise symptoms.

## Discussion

### Approaching symptom interpretation

Lack of theory within health services research often results in a step-by-step cookbook approach to methods [11]. This is also the case with current methodological approaches to patient delay, which, we will argue, largely work within the framework of empiricism, assuming that people are able to provide comparable answers of symptom experiences despite differences in social and cultural positions (Appendix 1).

In the study of patient delay various methodological approaches such as interviews, [12-14] and large surveys [4,15] have been applied. Studies estimating the prevalence of patient delay and its socio-demographic distribution are often large surveys, while studies exploring the causes of patient delay more frequently apply different methods. A minority of these are theoretically well based, often within the psychological tradition e.g. [3,16,17]. An often cited study exploring the causes of patient delay is Andersen, Cacioppo and Robert's psychophysiological comparison study of two groups of women seeking diagnostic evaluations of gynaecological and breast cancers. In this study they asked respondents to identify a series of calendar dates such as when they had first detected bodily changes, when they had first thought of it as potential illness, when they had decided to contact health professionals etc. Based on the empirical findings (and on earlier work by Safer et al.) they developed a model of total patient delay, comprising five different stages [3]. Such a detailed approach to patient delay is, however, uncommon. The strategy often pursued in large surveys is to estimate the patient delay period by asking two questions: When respondents first experienced the symptoms they consider are related to their cancer diagnosis and when they first contacted health professionals [4,5].

Regardless of methodological approach these, often otherwise exemplary studies, do not acknowledge the quite complex nature of how bodily sensations are recognised, interpreted and assigned meaning as symptoms, and how this affects the time estimates received.

Years of primarily sociological and anthropological research have revealed that the interpretation of bodily sensations as symptoms is embedded within a social and cultural reality e.g. [18-26]. In this literature a bodily sensation is a physiolocial experience, which may or may not be experienced as something significant such as a symptom of illness. That is, sensations never start as symptoms [18]. They only become symptoms post hoc, after an interpretation that they are abnormal. How people experience and understand sensations and how they are eventually interpreted as e.g. symptoms of a given cancer disease happens in relation to a specific social and cultural context. One of the pioneers presenting such ideas was Irving Kenneth Zola. In a line of frequently cited studies illustrating how symptoms evolve within a socio-cultural context he showed that diarrhoea, sweating and coughing were considered everyday bodily sensations among Mexican Indians of the South-western United States. In another study he showed that feelings of tiredness were not a cause of concern among students who value hard work [19,20]. In line with the work of Zola, the sociologist Angelo Alonzo developed a theoretical framework called the *situational approach*. In brief, it stresses the fact that symptoms are not only related to the individual, but evolve and are defined in specific situations. Alonzo defined the process by which this is done as '*containment*', meaning that bodily sensations are not defined as symptoms as long as they can be integrated within the individual's normal daily life [21]. Symptoms do not simply emerge as physiological realities, "rather they emerge from the interaction of biophysical sensations and the processes of social objectification or selection, interpretation and evaluation" [21]. The body express itself and is interpreted differently weather you are a football player resting after a rough game or a blue collar worker dozing off in the subway on the way home from work.

Zola's and Alonzo's work have since been extended by a line of sociologists and anthropologists that place an even stronger emphasis on the contextualised body [22-26]. Taken together these studies illustrate that bodily experience cannot be separated from elements such as cultural knowledge, social relations and social positioning, hence interpretative skills both apply meaning to sensations and fundamentally define how sensations are experienced [24,25]. In a recent study, Hay (2008) for example shows how identity management and social obligations influence how bodily sensations are converted into symptoms. For bodily sensations to be recognised as symptoms they need to be confirmed as such in the social arena, and how this is done depends partly on the individual's social position [18].

Other studies show more specifically how gender influences the symptom interpretation process. It has been indicated that the traditional female role encompasses more social responsibility in relation to the family, and that women therefore have a tendency to neglect and downplay the importance of their own health. Others hypothesise that gender-specific socialisation results in differences in bodily awareness; hence, women have a higher body awareness than men, which influences how they perceive bodily sensations [25,27].

These impositions against a too simplistic approach towards measuring patient delay could be confirmed by a line of psychological approaches often applied in exploring health related behaviour and care seeking. Several studies have highlighted the role of psychological factors, such as cognitive and emotional processes [28] or mood states [16] as important factors influencing how bodily sensations are experienced. In a classic study Pennebaker showed that physiological changes are experienced differently according to our involvement, or our committed engagement in what we are doing [29]. For example, touching a vibrating board will be experienced differently according to whether one has been told previously that one will experience a degree of either pleasure or of pain [24]. Andersen, Cacioppo and Roberts also touch upon the problem of measuring delay in their article on patient delay stages [3]. They even declare that "it may be more accurate to consider these data [the women's retrospectively elicited illness episodes and delay periods] as representing the individual's schema of the illness episode". Based on their empirical findings exploring patient delay among two groups of women with a cancer diagnosis and cancer-related symptoms respectively, they, however, end up concluding, that the emotional crisis following a cancer diagnosis 'did not impact the accuracy of recall' in the two groups. It could be argued that they do not fully draw the consequences of their own reasoning. If cognitive schemas influence symptom interpretation it might not be possible to detect differences among the two groups of women. The problem of estimating patient delay is, namely, not merely a problem of recall bias; rather it is a problem of symptom interpretation. Framing it as a problem of recall bias indicates that symptoms can be methodologically approached as objective clinical realities whose time of appearance can easily be measured; an approach that does not fully consider the fact that symptoms evolve in relation to a specific social and cultural context, and perhaps a specific psychological state (Appendix 2).

### What are we measuring in patient delay studies?

Notwithstanding the particular theoretical perspective taken, substantial research exemplifies that we cannot, as currently done in many patient delay studies, assume that respondents define delay periods in identical ways, nor can we infer that the periods reported inform us on how long care seeking was delayed in an empiricist understanding of the concept. But what are we measuring, then? Following the line of argument presented above, we are perhaps merely measuring differences in response to bodily sensations and symptoms, and how these are linked to specific cancer sites. As touched upon many patient delay studies have sought to identify socio-demographic variables associated with patient delay [4,30,31]. Hypothetically, what they are, in fact, identifying could be socio-demographic differences in indicators of how people establish the link between bodily sensation, symptom and diagnosis, and not the variables predicting delay. In the same manner, studies exploring psychological factors such as emotions or the effect of cognitive schemas on symptom interpretation could merely be exploring psychological differences in symptom interpretation. In epidemiological terms, one could argue that the 'baseline meaning' attached to bodily sensations and how they are converted into symptoms related to a specific cancer diagnosis is not the same for all individuals; hence, the delay periods may not be comparable. The socio-cultural context or the psychological factors may be 'confounders' in establishing the relation between bodily sensation, symptom and diagnosis when estimating patient delay.

## Future research

The discussion of how we measure patient delay is, thus not new, hence it relates to a wider discussion of how we deal with cultural and processual factors in epidemiology [32]. A discussion which was also brought forward by Zola in one of his early works, where he noted that in any community unexplained epidemiological differences may be due more to the differential occurrence of these factors which reflect the "selectivity and attention which get people and their episodes into medical statistics, rather than any true difference in the prevalence and incidence of a particular problem or disorder" [32].

Consequently, when studying patient delay, validity will always be a matter of degree. There is no simple recipe for establishing evidence of validity on this issue, being it in relation to cancer or any other illness [33]. Following the line of reasoning presented above it could even be argued that it - epistemologically speaking - is an impossible task. From a medical perspective patient delay is, however, an important issue that receives much attention. Embarking on methodological discussions like these will potentially help us improve future patient delay measures and raise a general awareness on 'our wrong-doings' when approaching complicated issues such as symptom interpretation. The methodological challenges posed by the theoretical approaches on symptom interpretation, we will argue, call for alternative means of data collection in the middle ground between traditional anthropological or sociological research and medicine. In order to do to so, we suggest that research is enhanced on three accounts:

1) Research on symptom interpretation processes

2) Research on how patient delay may be operationalised

3) Research on the development of valid instruments for measuring patient delay

### Understanding symptom interpretation processes

In order to fully understand patient delay and how people define the time intervals they are asked to report, we need to understand the interpretive processes that lie prior to care seeking. One avenue could be to conduct prospective studies on symptom interpretation and care seeking that are theoretically grounded within disciplines such as psychology, anthropology and sociology; disciplines that have traditionally worked with the relation between meaning making (symptom interpretation) and practice (seeking care) [22]. Besides providing us with new, valuable forms of knowledge, this perspective would improve data validity. The vast majority of patient delay studies are retrospective. Informants have already become patients and the data material we achieve depends on how they recall and legitimize their decision to seek care [18,34]. Symptom interpretation is embedded within a given socio-cultural context, which - as argued - may result in the reporting of non-comparable measures of delay. However, the socio-cultural context also poses limitations and constrains in relation to how informants report on symptom experiences. A line of studies show that illness episodes are embedded in social definitions of proper attitudes, actions and activity levels [34,35]. This will be reflected in informants' representations of symptom interpretation and seeking decisions. In epidemiological terms one could say that the socio-cultural context modulates responses given. An aspect which is briefly touched upon in the case of Mr. A. (Appendix 3). Retrospectively reported material does, therefore, not necessarily give us insight into how people initially experienced bodily sensations or interpreted these as symptoms needing professional care.

Furthermore, prospective studies could potentially provide a more differentiated perspective on how decisions to seek care are made. When symptoms are interpreted as objective clinical realities, as is the case within the empiricist tradition, we not only disguise the more complex processes through which bodily sensations are recognised as symptoms that needs professional care, we also limit patient delay studies to simple assessments of whether the patients were competent in recognising signs of potential disease [18]. Interpreting bodily sensations as symptoms that need care seeking is, however, not merely a matter of competence vs. incompetence. Firstly, because bodily sensations are interpreted as symptoms in relation to the individual patient's specific situation (What caused tiredness? What caused rectal bleeding?). Secondly, as will be discussed below, because it is equally difficult from a medical perspective to firmly establish relations between specific symptoms and cancer.

### Operationalising patient delay

Patient delay is only relevant in relation to symptoms for which there is an established relationship to the presence of cancer. To be able to conduct patient delay studies we need to know as precisely as possible how specific signs and bodily sensations (e.g. blood in the stool or tiredness) are related to specific cancer types. For many signs and bodily sensations such a relationship is not well established and more research is required in populations from general practice. Recent research thus indicates that lower urinary tract symptoms are not related in any way to the presence of prostate cancer [36,37]. It can therefore be questioned if Mr D is right when he reported 6 month of delay or Mr C was more correct in reporting no delay (Appendix 4).

Today, patient delay estimates are based on patients reporting about presence of symptoms. I.e. when they interpreted bodily sensations as a sign of the cancer they later was diagnosed with. Our estimate of delay thus depend on the presence of a specific sign or sensation and at the same time the patients interpretations of this. We need to separate these two conceptually and operationalise them as accordingly. If we are interested in the presence or absence of a specific sign or sensation we must downplay the patient's interpretation. This may improve validity as the patient delay estimates would become more comparable, and it would establish a common ground among researchers conducting delay studies. In the case of Mr A this would have established the presence of blood in the stool for the first time 4 month before the time he spontaneously reported as the first time he had a sign of his cancer (Appendix 3).

### Measuring patient delay

Once we have operationalised patient delay and established knowledge about symptom interpretation processes, we must develop valid measures. We suggest that efforts be devoted to the development of two different methodological approaches that both feed on the above mentioned research.

#### Interviews

The validity of patient delay measures could potentially be improved if detailed information on patient experienced symptoms were gathered through more personal approaches (e.g. structured interviews/telephone interviews, during clinical encounters) [5]. The estimates could potentially be improved if patient interpretations were downplayed and the delay period was estimated by health professionals after the interview/clinical encounter and based on standard clinical evaluations of the relation between symptoms and specific cancers as mentioned above. As mentioned, patient delay estimates provided by patients are not comparable, hence the answers provided are embedded within a given socio-cultural context. This is of course not overcome simply by letting health professionals estimate delay periods, however a health professionally driven approach may allow for a more systematic comparison of symptom experiences, if done based on e.g. the above mentioned clinically relevant operationalisations.

#### Questionnaires

**Figure 1:**
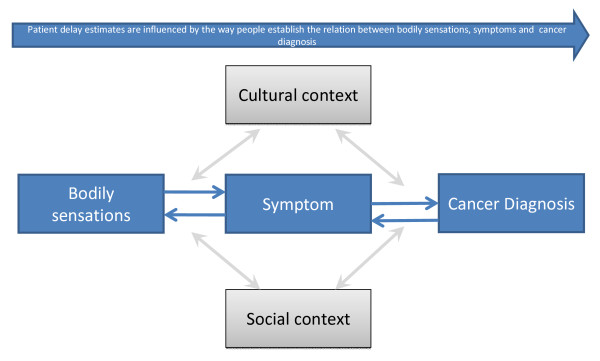
**A simple model of the relation between bodily sensations, symptoms and patient delay**. The figure illustrates how the symptom interpretation process affects how people estimate their delay periods.

Furthermore, we could benefit from doing research into 'contextual confounders' or 'psychological confounders' of patient delay. How does the socio-cultural context influence the symptom interpretation process and, hence, how do people report delay periods? (Figure 1). If, as indicated, women experience bodily sensations differently than men due to a specific cultural socialisation and positioning, how does this affect how they report delay periods? If it is possible to identify social and cultural patterns in symptom interpretation, such identification could form the basis for developing valid measures (e.g. postal questionnaires), as it would be possible to adjust for the socio-cultural confounders. Research on symptom interpretation processes could be appropriate in this regard.

## Summary

Current studies on patient delay do not sufficiently consider the fact that symptoms are social constructs. As illustrated, there is no simple relation between bodily sensation, symptom and cancer diagnosis, and we cannot assume that the respondents report delay periods in identical ways. Consequently, the delay periods reported by respondents do not necessarily inform us on how long they actually delayed care seeking. Rather, we may merely be measuring differences in response to bodily sensations and symptoms, and how these are linked to specific cancer sites. In order to improve the validity of patient delay studies, it is suggested that we improve our research on three fronts: More research should be devoted to symptom interpretation processes, more research should target how to operationalise patient delay, and, importantly, more research is needed on how to develop valid instruments for measuring patient delay

## Competing interests

The authors declare that they have no competing interests.

## Authors' contributions

RSA has been in charge of developing the main arguments of the article and written the final manuscript.

JS, FO, FB and PV have made substantial contributions to developing the arguments of the article, critically revised the article for important intellectual content, and read and approved the final version of the manuscript.

## Appendix 1 Two different methodological approaches to patient delay

### Current empiricist approach to patient delay

It is possible to measure the time from symptom onset until care seeking, because there is a direct link between a symptom and the physiological disorder.

It is assumed that all respondents define delay periods in identical ways.

### Contextual approach to patient delay

It is complicated to measure the time from symptom onset until care seeking, because the interpretation of bodily sensations as symptoms related to a specific physiological disorder is embedded within the individual's social and cultural reality.

It cannot be assumed that all respondents define delay periods in identical ways.

## Appendix 2 The symptom interpretation process summarized

Bodily sensations are embodied; they are felt experiences. In order to be defined as symptoms, they undergo an interpretive process which is influenced by the individual's socio-cultural context.

How and if bodily sensations such as rectal bleeding, coughing, tiredness and nocturia are interpreted as symptoms is affected by the individual's social situation (e.g. am I tired because my mother is ill? Am I coughing because of the dust at my work?) and the wider cultural context (e.g. gender specific socialisation, social relations and obligations). Hence symptoms are social constructions.

When we are studying patient delay, patients are asked to define when the symptoms related to their cancer evolved. That is, we ask them to establish the relation between:

bodily sensation ↔ symptom ↔ cancer diagnosis

How people establish these relations is influenced by their specific social and cultural context and will differ from individual to individual. Consequently, the delay periods reported are not comparable.

## Appendix 3: The contextual perspective exemplified

In order to illustrate the problems of measuring patient delay raised by the contextual perspective, we present a series of cases from a project on patient delay currently carried out at the Research Unit for General Practice, University of Aarhus. The examples illustrate how differences in symptom interpretation influence the delay periods reported.

### Mr. A interviewed on his experiences with his colon cancer patient

Mr. A reported that he had experienced rectal bleeding and upset stomach for about two months prior to care seeking. During the interview, he explained that he had first thought of it as 'stomach flu', "which I thought would just pass, but now I can see it was probably the cancer that had begun." His wife also participated in the interview, and hearing her husband's explanation, she added: "Well, I think it goes well beyond that. It is more than two months. I know, because I saw blood in your panties long before we went on summer holiday, which is at least four months ago. I was alarmed, but you would not listen." They began a discussion on when his symptoms had actually started, and he suddenly remembered having experienced rectal bleeding around Christmas, nine months prior to the care seeking event. When asked what he had thought of the rectal bleeding, he said that he had thought of it as haemorrhoids, which he had suffered from a few years earlier.

### Mrs. B interviewed on her experiences with her lung cancer

When asked when she had first experienced symptoms of her cancer, Mrs. B answered: "Well, I do not know, I do not know if it was four years ago or just one year ago." Mrs. B smokes cigarettes and four years prior to her cancer, she had been diagnosed with what she called 'early COPD'. All those years she has suffered from coughing and continuous colds. "Also, sometimes it felt like there was something I could not cough up, you know; it sort of stuck to my lungs. But I just thought it was the early COPD. Now, I can see that it was probably the cancer all along. It has probably been there for years." Her symptom interpretation was also influenced by her family situation. Within the last six months before the care seeking event, her father had died and her mother had fallen ill; a situation which also influenced the way she had understood her symptoms. "I was really not paying attention to myself these months. And the tiredness, I experienced, well I thought it was just because of all this [her family situation]."

The cases of Mr. A and Mrs. B clearly illustrate how people experience and struggle with interpreting bodily sensations within the context of their daily life, and how this adds to the complexity of identifying these sensations as potential cancer symptoms. In both cases, their social situations and competing diseases established a platform of interpretation producing divergent explanations. Mr. A had earlier suffered from haemorrhoids, which provided him with a legitimate way of defining his symptoms as 'not worrisome'; a perspective that was clearly not shared by his wife, who had different perspectives on symptom interpretation. According to his wife, Mr. A was too hesitant recognising symptoms of illness. The case presented does not reveal why, but other parts of the interview indicate that illness, to Mr. A, connotes weakness, which contradicts his sense of self as a strong and active man; a fact which may have influenced how he actually interpreted his first experiences of rectal bleeding, but also how he chose to present them.

It was also difficult for Mrs. B to establish the link between her cancer and her experiences of how her body had changed. Sometimes she interpreted her coughing and her tiredness as symptoms of her COPD, at other times she figured it was due to the difficulties of her family situation.

## Appendix 4 The contextual perspective exemplified

### Mr C interviewed on his experiences with his prostate cancer

Mr. C reported that he had not had symptoms of his cancer prior to the diagnosis. During the interview, however, he explained that he had suffered from frequent nocturia for approximately two years prior to the care seeking event. The problem had increased in intensity during the last year. When asked what he thought might be the cause of this, he said: "well, this is normal for a man at my age. You know, when a man turns fifty he enters the 'the night-pissing team'. I thought it was just old age, and therefore I did not think of it as anything."

### Mr D interviewed on his experiences with his prostate cancer

Mr. D reported that he had experienced symptoms of his cancer for six months prior to care seeking. During the interview it became clear that he had experienced nocturia for at least two years. When asked what he had thought of this, he gave the same explanation as Mr C; that men urinate more often when they grow older. However, during the last six months it had worried him, and he felt that he was 'going more often.' Therefore, he estimated that he had had symptoms for about six months. "And that was also the period where my wife started noticing, and then I thought it might mean something."

In hindsight, the symptom experiences of Mr. C and Mr. D are similar. They both experienced nocturia for two years. However, the cases reveal that differences in *when *they interpreted bodily sensations (nocturia) as symptoms resulted in the reporting of dissimilar delay periods (no delay and six months of delay). Both cases illustrate that lay-understandings and gender-specific expectations of how bodily functions alter with age influenced the respondents' interpretations.

## Pre-publication history

The pre-publication history for this paper can be accessed here:

http://www.biomedcentral.com/1472-6963/9/189/prepub
